# ULK1 phosphorylates Exo70 to suppress breast cancer metastasis

**DOI:** 10.1038/s41467-019-13923-7

**Published:** 2020-01-08

**Authors:** Liyuan Mao, Yan-yan Zhan, Bin Wu, Qiang Yu, Liang Xu, Xiaoting Hong, Linhai Zhong, Panying Mi, Li Xiao, Xinquan Wang, Hanwei Cao, Wenqing Zhang, Binbin Chen, Jingzhou Xiang, Kunrong Mei, Ravi Radhakrishnan, Wei Guo, Tianhui Hu

**Affiliations:** 1 Cancer Research Center, School of Medicine, Xiamen University, 361102 Xiamen, China; 2 Department of Biology, University of Pennsylvania, Philadelphia, PA 19104 USA; 3 Department of Oncology, Zhongshan Hospital Affiliated to Xiamen University, 361004 Xiamen, China; 4 School of Life Sciences, Tsinghua University, 100084 Beijing, China; 5 School of Pharmaceutical Science and Technology, Tianjin University, Tianjin, China; 6 Department of Bioengineering, School of Engineering, University of Pennsylvania, Philadelphia, PA 19104 USA

**Keywords:** Biochemistry, Cancer, Cell biology

## Abstract

Increased expression of protein kinase ULK1 was reported to negatively correlate with breast cancer metastasis. Here we report that ULK1 suppresses the migration and invasion of human breast cancer cells. The suppressive effect is mediated through direct phosphorylation of Exo70, a key component of the exocyst complex. ULK1 phosphorylation inhibits Exo70 homo-oligomerization as well as its assembly to the exocyst complex, which are needed for cell protrusion formation and matrix metalloproteinases secretion during cell invasion. Reversely, upon growth factor stimulation, Exo70 is phosphorylated by ERK1/2, which in turn suppresses its phosphorylation by ULK1. Together, our study identifies Exo70 as a substrate of ULK1 that inhibits cancer metastasis, and demonstrates that two counteractive regulatory mechanisms are well orchestrated during tumor cell invasion.

## Abstract (web summary)

Elevated expression of ULK1 is known to be inversely correlated with breast cancer metastasis. Here, the authors report Exo70 as a substrate of ULK1 that suppresses cancer metastasis, and show that ERK1/2 mediated phosphorylation of Exo70 leads to opposing effects on tumour cell invasion.

## Introduction

Breast cancer is among the most prevalent cancers and the first leading cause of cancer-related deaths in women globally^1^. Despite recent advances in breast cancer diagnosis and treatment, the mortality rate remains high due to the regional and distant dissemination after the primary tumor resection and the failure of the current anti-tumor therapies in inhibiting metastasis. There is an urgent unmet need to understand the molecular players and the regulatory mechanisms involved in the metastasis of breast cancer and develop better therapeutic strategies to block metastasis.

ULK1 (Unc-51-like kinase 1), an evolutionarily conserved serine/threonine kinase, plays a central role in the autophagy pathway to redirect cellular resources in response to stress^2–4^. ULK1 was recently linked to the metastasis of several types of tumors, such as non-small-cell lung cancer, esophageal squamous cell carcinoma, and breast cancer^5–8^. The phosphorylation of ULK1 on Ser757 inactivates ULK1, and is correlated with poor survival in non-small-cell lung cancer patients^5^. It was shown that, under metabolic stress, ULK1 phosphorylates the focal adhesion kinase (FAK) family interacting protein of 200 kDa (FIP200), which subsequently leads to the inhibition of FAK-directed tumor cell motility and metastasis in non-small cell lung cancer^5^. In breast cancer, low expression of ULK1 was found to associate with lymph node metastasis and poor patient survival^7^. Suppression of autophagy in the hypoxic tumor microenvironment, using hypoxia-induced expression of the dominant-negative mutant of ULK1 or ATG4B, increased fibronectin deposition and facilitated breast cancer cell migration^8^. However, the role of ULK1 and the mechanisms involved in the regulation of breast cancer metastasis are still poorly understood.

The exocyst is an evolutionarily conserved octameric complex consisting of Sec3, Sec5, Sec6, Sec8, Sec10, Sec15, Exo70, and Exo84. The exocyst mediates the tethering of secretory vesicles to the plasma membrane during exocytosis^9–12^. Recently, a role for the exocyst in cell migration and invasion has been reported^13–24^. The exocyst subunit Exo70 interacts with the Arp2/3 complex, a key nucleator of actin assembly, and enhances its binding to the nucleation-promoting factor WAVE2, thereby promoting lamellipodia formation and directionally cell migration^17,21,25^. Knocking down endogenous Exo70 in cancer cells significantly decreased their invasion, owing to the weakened actin polymerization and a block in the secretion of matrix metalloproteinases (MMPs)^14,17,18,24^. In addition to interacting with the Arp2/3 complex, Exo70 proteins also form homopolymers that induce membrane curvature for membrane protrusion formation^25^.

Here we report Exo70 as a direct phospho-target of ULK1. Phosphorylation of Exo70 by ULK1 inhibits the metastasis of breast cancer cells. Mechanistically, ULK1 phosphorylation of Exo70 inhibits its homo-oligomerization and assembly into the exocyst complex, thereby blocking the formation of cell protrusion and the secretion of MMPs. Interestingly, the inhibitory phosphorylation by ULK1 is counteracted by extracellular signaling-regulated kinase (ERK), which phosphorylates Exo70 at a different site upon growth factor signaling. Our study delineates a molecular pathway by which ULK1 regulates tumor invasion through direct phosphorylation of Exo70, and also reveals a counteractive regulatory mechanism that functions during tumor invasion.

## Results

### ULK1 inhibits breast cancer metastasis

**Fig. 1:**
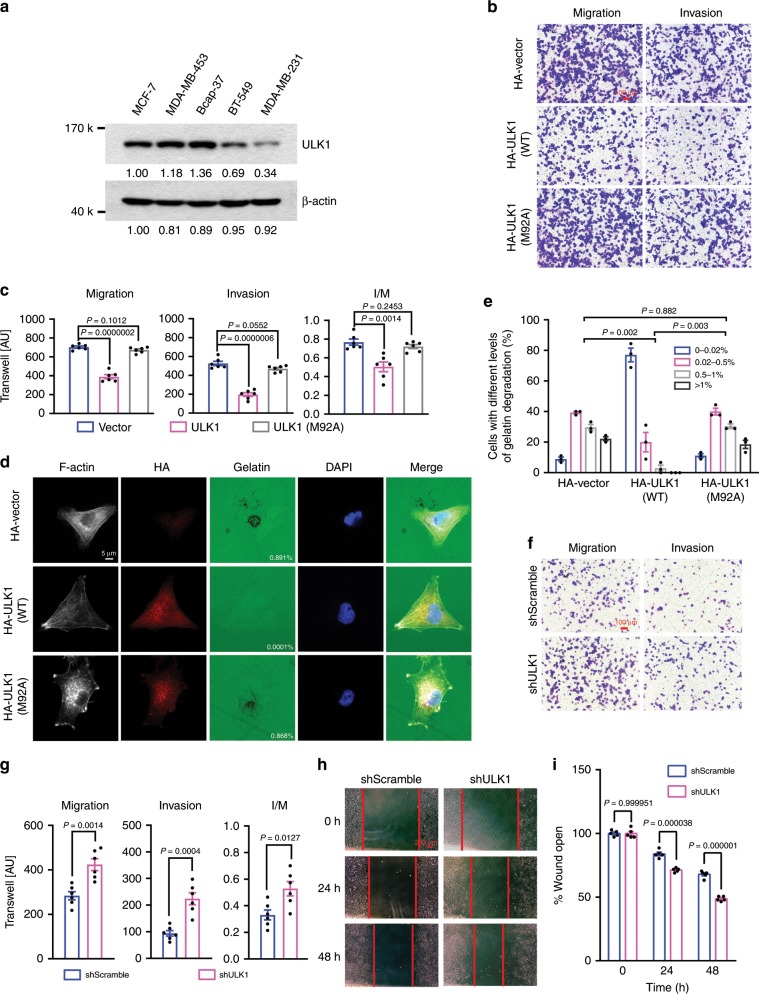
ULK1 inhibited migration and invasion of breast cancer cells. **a** The levels of ULK1 expression in breast cancer cell lines of different metastasis ability were examined by western blotting. β-Actin was used as a loading control. **b**, **c** Effects of ULK1 and ULK1(M92A) on the migration and invasion of MDA-MB-231 cells. Transwell assay was carried out using cells transfected with empty vector, HA-ULK1, or HA-ULK1(M92A) plasmid. Representative images (scale bar: 100 μm) (**b**) and cell counts (**c**) were shown (*n* = 6 biologically independent samples). AU: arbitrary unit; I/M: ratio of invasion (I) to migration (M). **d**, **e** Overexpressed ULK1 but not ULK1(M92A) significantly inhibited the degradation of extracellular matrix by MDA-MB-231 cells. Representative images (**d**) and gelatin degradation levels from 45 cells (**e**) for each group were shown (*n* = 3 independent experiments). Scale bar: 10 μm. **f**–**i** ULK1 knockdown increased the invasion and migration ability of MCF-7 cells as examined by the transwell assay (**f**, **g**) (scale bar: 100 μm) and wound healing assay (**h**, **i**) (scale bar: 200 μm). Representative images (**f**, **h**), cell counts (**g**), and quantification of wound distance (**i**) were shown (*n* = 6 biologically independent samples for **f**, **g**, *n* = 5 biologically independent samples for **h**, **i**). Data represented the means ± SEM. *P* values were analyzed by unpaired two-tailed Student’s *t* test and Kruskal–Wallis test (**e**).

To investigate the role of ULK1 in breast cancer metastasis, we examined the expression level of ULK1 in several human breast cancer cell lines with different metastatic potential. ULK1 expression was lower in the highly metastatic cell lines (MDA-MB-231 and BT-549) than those in the low metastatic cell lines (MCF-7, MDA-MB-453, and Bcap-37) (Fig. 1a), suggesting that ULK1 expression is negatively correlated to the metastatic potentials of human breast cancer cells. We then analyzed the effect of ULK1 on invasion. Overexpression of ULK1, but not the kinase-dead mutant, ULK1(M92A)^26^, significantly inhibited migration and invasion of the metastatic MDA-MB-231 cells without affecting their growth or survival during the observed period (Fig. 1b, c and Supplementary Fig. 1a, b). Moreover, overexpression of ULK1 but not ULK1(M92A) significantly inhibited invadopodium-mediated matrix degradation by MDA-MB-231 cells (Fig. 1d, e). Reversely, knocking down ULK1 in the low metastatic cell line MCF-7 increased its invasion and migration ability as indicated by the transwell assay and wound healing assay (Fig. 1f–i and Supplementary Fig. 1c–e).

### Exo70 interacts with and is phosphorylated by ULK1

**Fig. 2:**
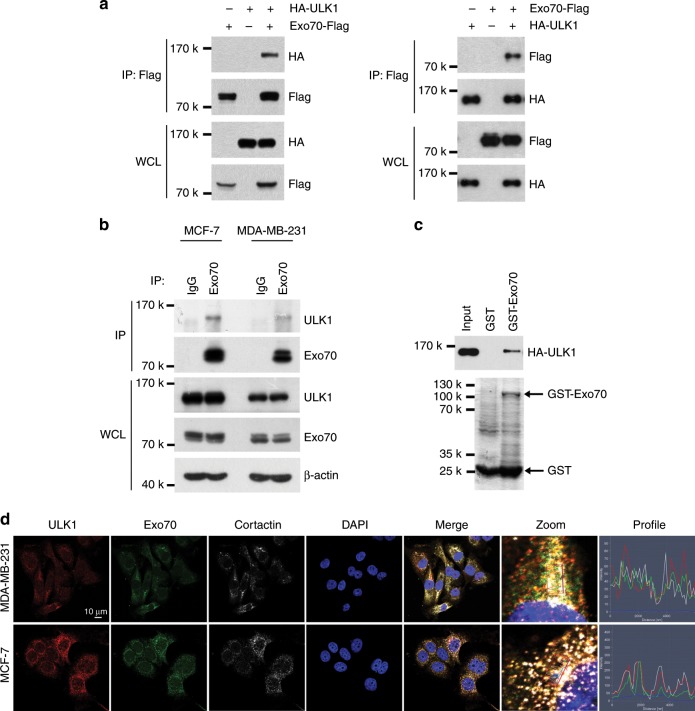
Exo70 interacts with ULK1. **a** Interaction of Exo70 and ULK1 in 293T cells was detected by co-immunoprecipitation assay. Whole-cell lysates (WCLs) and immunoprecipitated (IP) proteins were analyzed by western blotting. **b** The interaction of endogenous Exo70 with ULK1 in MCF-7 and MDA-MB-231 cells were examined by co-immunoprecipitation using anti-Exo70 or anti-IgG (negative control) antibody. **c** The interaction of Exo70 and ULK1 was determined by GST pull-down assay. Purified HA-ULK1 protein was incubated with recombinant GST-Exo70 protein conjugated to the Glutathione Sepharose. GST-Exo70 was examined by Coomassie Blue staining (lower panel) and the bounded HA-ULK1 was detected with anti-HA antibody (upper panel). GST was used as a negative control. **d** The co-localization of endogenous Exo70, ULK1, and cortactin was demonstrated with immunofluorescence in MCF-7 and MDA-MB-231 cells. Scale bar: 10 μm.

To investigate how ULK1 suppresses breast cancer metastasis, immunoprecipitation (IP) assay in cells expressing HA-tagged ULK1 was carried out. Candidate ULK1-interacting proteins co-precipitated with HA-ULK1, but not in that of the vector control, were analyzed by mass spectrometry (MS). Exo70, a subunit of the exocyst complex, was identified in the assay. The ULK1-Exo70 interaction was first confirmed by IP assays using tagged proteins exogenously expressed in cells (Fig. 2a) and then with endogenous proteins in different breast cancer cell lines including MDA-MB-231 and MCF-7 (Fig. 2b). Furthermore, bacterially expressed GST-Exo70 binds to purified HA-ULK1 (Fig. 2c). Co-localization of ULK1 with Exo70 in dynamic actin filament networks critical for membrane trafficking and remodeling as indicated by cortactin counterstaining was observed by immunofluorescence microscopy in MCF-7 and MDA-MB-231 cells (Fig. 2d).

**Fig. 3:**
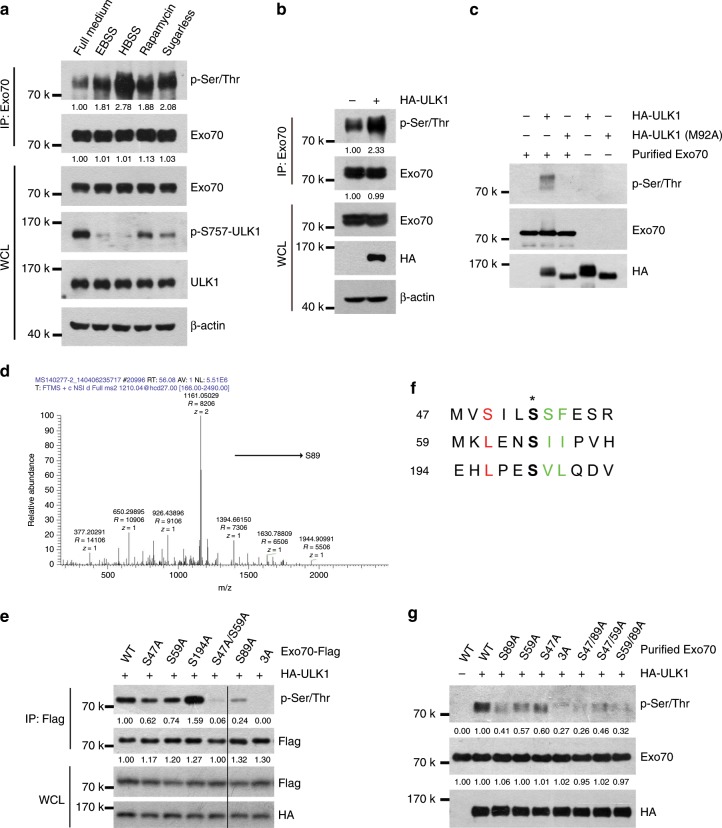
Exo70 is phosphorylated by ULK1 on Ser47, Ser59, and Ser89. **a** The level of threonine/serine phosphorylation of endogenous Exo70 under starvation conditions or rapamycin treatment. MDA-MB-231 cells were placed in starvation medium (EBSS, HBSS, or DMEM medium sugarless) or treated with rapamycin (50 nM) for 2 h, and then subjected to immunoprecipitation using anti-Exo70 antibody. Whole-cell lysates (WCLs) and immunoprecipitated (IP) proteins were analyzed by western blotting. Threonine/serine phosphorylation on Exo70 was detected by anti-p-Ser/Thr antibody. **b** 293T cells were transfected with empty vector or HA-ULK1 plasmid, and immunoprecipitation assay was carried out with anti-Exo70 antibody. Overexpression of ULK1 (HA-ULK1) increased the threonine/serine phosphorylation of endogenous Exo70 protein. **c** In vitro phosphorylation of Exo70 by ULK1. Bacterially purified Exo70 was incubated with purified HA-ULK1 and HA-ULK1(M92A) in the in vitro phosphorylation assay as described in Materials and methods. **d** Potential ULK1-phosphorylated sites within the N terminus of Exo70 based on the consensus ULK1 phospho-substrate sequence. **e** Effects of Exo70 mutations on its phosphorylation induced by ULK1. HA-ULK1 was co-transfected with the indicated FLAG-Exo70 plasmids in 293T cells, and then subjected to immunoprecipitation with anti-Flag antibody. Phosphorylation of Exo70 at serine/threonine was examined with p-Ser/Thr antibody. **f** Ser89 was identified to be another potential ULK1-phosphorylated site using immunoprecipitation in combination with mass spectrometry (IP-MS). **g** In vitro phosphorylation of wild-type and mutant Exo70 by purified ULK1. Exo70 at serine/threonine was examined with p-Ser/Thr antibody.

We next investigated whether ULK1 phosphorylates Exo70. When cells were cultured in starvation medium or treated with rapamycin, ULK1 was activated as indicated by the decreased phosphorylation levels of ULK1 on Ser757. The levels of threonine/serine phosphorylation of endogenous Exo70 increased (Fig. 3a). In additi on, overexpression of ULK1 in cells grown in full medium^27^ increased the phosphorylation of Exo70 (Fig. 3b). To determine whether ULK1 directly phosphorylates Exo70, we conducted in vitro assay using recombinant Exo70 and purified ULK1. Exo70 was phosphorylated by ULK1, but not the kinase-dead ULK1(M92A) mutant protein (Fig. 3c). Together, these data strongly suggested that Exo70 is a phospho-substrate of ULK1.

Next, the ULK1 phosphorylation sites in Exo70 were analyzed. Exogenous Exo70 was purified by IP in ULK1 overexpression cells, and then subjected to MS. Ser89 of Exo70 was identified as a potential ULK1 phosphorylation site (Fig. 3d). The Exo70(S89A) mutant was then generated and expressed alone or with ULK1 in 293T cells. The S89A mutation dramatically diminished, but not eliminated, the phosphorylation of Exo70 induced by ULK1 (Fig. 3e), suggesting that Ser89 was indeed crucial for ULK1 to phosphorylate Exo70, but some other ULK1-phosphorylated sites on Exo70 also exist. According to the consensus ULK1-substrate motif^28^, three other possible ULK1-phosphorylated sites (Ser47, Ser59, and Ser194) were identified at the N terminus of Exo70 (Fig. 3f). ULK1-induced phosphorylation of Exo70 was moderately suppressed by the single-point mutants S47A or S59A, but not S194A (Fig. 3e). Moreover, Exo70 phosphorylation by ULK1 was greatly suppressed by the double mutant S47A/S59A. Triple mutation on Exo70 (Exo70-3A: S47A/S59A/S89A) blocked its phosphorylation (Fig. 3e). The in vitro phosphorylation assays demonstrate that Ser47, Ser59, and Ser89 were responsible for the phosphorylation of Exo70 directly catalyzed by ULK1 (Fig. 3g). Ser47, Ser59, and Ser89, with their flanking sequences, are highly conserved in eukaryotic cells (Supplementary Fig. 2).

### ULK1 phosphorylation of Exo70 inhibits metastasis

**Fig. 4:**
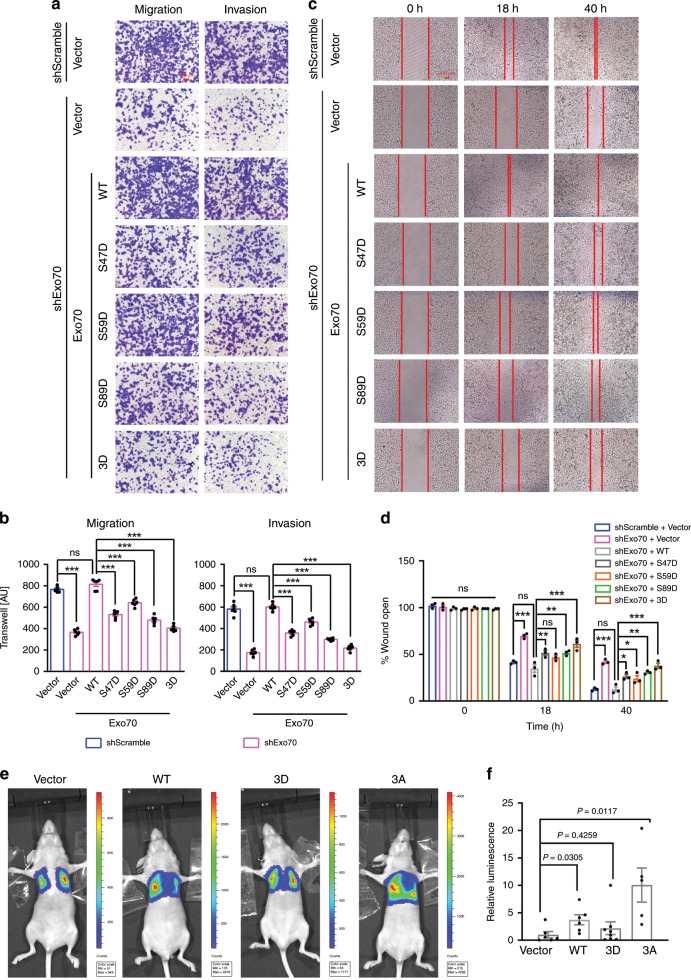
Phosphorylation of Exo70 on Ser47/59/89 inhibited breast cancer metastasis. **a**–**d** Exo70 phosphorylation on Ser47, Ser59, and Ser89 inhibited the migration and invasion of MDA-MB-231 cells as analyzed by transwell assay (**a**, **b**) (scale bar: 100 μm) and wound healing assay (**c**, **d**) (scale bar: 200 μm). Control (Scramble) and Exo70 shRNA knockdown cells were transfected with empty vector, Exo70-Flag or Exo70 mutant variants. Representative images (**a**, **c**), cell counts (**b**), and quantification of wound distance (**d**) were shown (*n* = 6 biologically independent samples for **a**, **b**, *n* = 3 biologically independent samples for **c**, **d**). AU: arbitrary unit. **e**, **f** Exo70 phosphorylation on Ser47, Ser59, and Ser89 suppressed the metastasis of MDA-MB-231 cells to the lung. MDA-MB-231 cells stably expressing empty vector, Exo70-Flag, Exo70(3D)-Flag, or Exo70(3A)-Flag were subjected to tail-vein injection into nude mice (vector *n* = 6, WT *n* = 6, 3D *n* = 8, 3A *n* = 5). Representative images (**e**) and quantification (**f**) of Luciferase expression within the lungs of nude mice in each group were shown. Data represented the mean ± SEM. The difference in significance was analyzed by one-way analysis of variance (ANOVA) (**b**, **d**) or unpaired two-tailed Student’s *t* test (**f** no adjustment was made for multiple comparisons). *P < 0.05; **P < 0.01; ***P < 0.001; and n.s., nonsignificant vs. the indicated control.

To test whether ULK1 suppresses breast cancer metastasis through phosphorylating Exo70, we analyzed the effect of Exo70 phosphorylation at Ser47, Ser59, and Ser89 on breast cancer metastasis. Three phospho-mimetic single mutants (Exo70-S47D, Exo70-S59D, and Exo70-S89D, respectively) and a triple mutant (Exo70-3D: S47D/S59D/S89D) were constructed and transfected individually into Exo70-knockdown MDA-MB-231 cells. Results from both transwell assay and wound healing assay showed that knockdown of Exo70 significantly decreased invasion and migration of MDA-MB-231 cells, and the effect was rescued by re-introducing the wild-type (WT) Exo70 (rat Exo70, which is not targeted by human shExo70). The expression of the single-point mutant S47D, S59D, or S89D, or the Exo70-3D triple mutant either partially or failed to rescue the inhibitory effect (Fig. 4a–d and Supplementary Fig. 3a). To further test the effect of ULK1-mediated Exo70 phosphorylation on cell migration and invasion, we carried out rescue experiments with the phospho-deficient Exo70-3A mutant. Overexpression of ULK1 inhibited the migration and invasion of Exo70-knockdown MDA-MB-231 cells, which express Exo70-WT but not Exo70-3A (Supplementary Fig. 3c). During the period of assay, cell number did not show significant difference among all the groups (Supplementary Fig. 3b, d).

To study the effect of ULK1 phosphorylation in vivo, metastasis assay was carried out in mice. Four MDA-MB-231 cell lines stably expressing Exo70-WT, Exo70-3D, Exo70-3A, or a control vector were injected into the tail vein of nude mice, and the pulmonary metastasis of these groups was detected and compared 4 weeks later using bioluminescence analysis. Overexpression of Exo70-WT and Exo70-3A, but not Exo70-3D, significantly promoted the pulmonary metastasis of breast cancer cells when compared to the control group. Notably, the Exo70-3A group had significantly higher level of metastasis even when compared to the Exo70-WT group (Fig. 4e, f and Supplementary Fig. 3e). No significant difference in the growth of xenograft tumors for these four cell lines was detected (Supplementary Fig. 3f–g). These data thus suggest that ULK1 phosphorylation of Exo70 inhibits breast cancer metastasis.

### ULK1 phosphorylation inhibits Exo70 oligomerization

**Fig. 5:**
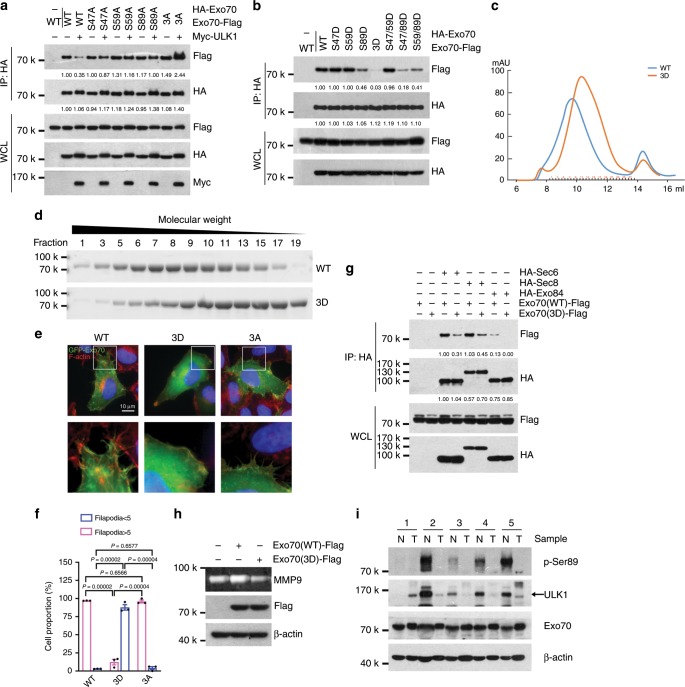
ULK1 phosphorylation suppressed Exo70 oligomerization. **a** The 293T cells were co-transfected with HA-tagged and Flag-tagged wild-type and mutant Exo70 in the presence or absence of Myc-ULK1, and then subjected to co-immunoprecipitation assay. **b** Self-interaction of Exo70 proteins was inhibited by its phosphorylation on Ser47, Ser59, and Ser89. The 293T cells were co-transfected with HA- or Flag-tagged Exo70 or its mutant variants, and then subjected to co-immunoprecipitation assay. **c** Recombinant Exo70 protein was loaded onto a Superdex 200 10/300GL column and the elution was analyzed. The elution peak of Exo70-3D shifted to a lower-molecular weight position compared to Exo70-WT. **d** Purified recombinant Exo70 protein was loaded onto a Superdex 200 10/300GL column and the eluted fractions were analyzed by SDS-PAGE and Coomassie blue staining. **e** Exo70-WT and Exo70-3A induced much more protrusions than Exo70-3D. MDA-MB-231 cells were transfected with GFP-Exo70, GFP-Exo70(3D), or GFP-Exo70(3A), and then stained with Rhodamine-phalloidin to label F-action. Scale bar: 10 μm. Magnification of boxed area was shown in the lower panel. **f** Protrusions index from at least 31 cells for each group was shown, distinguished by whether the number of filopodia in each cell is greater than five. Data represented the mean ± SEM (*n* = 3, three independent experiments), and the *P* values were analyzed by unpaired two-tailed Student’s *t* test. **g** The 293T cells were transfected with Exo70-Flag or Exo70(3D)-Flag, together with HA-Sec6, HA-Sec8, or HA-Exo84, and then subjected to co-immunoprecipitation assay. Interaction of Exo70 with Sec6, Sec8, and Exo84 were inhibited by phosphorylation of Exo70 on Ser47, Ser59, and Ser89. **h** Effects of Exo70 mutations on MMP-9 secretion in MDA-MB-231 cells as analyzed by zymography. Cells were transfected with empty vector, HA-Exo70, or HA-Exo70(3D) plasmid. Cellular proteins were also prepared and subjected to western blotting, with β-actin as an internal control. **i** The levels of Exo70 phosphorylation on Ser89 and the expression levels of ULK1 in five pairs of primary breast tumor tissues and the matched adjacent normal tissues. β-Actin was used as an internal control. N, normal; T, tumor.

It was previously shown that the homo-oligomerization of Exo70 led to the induction of membrane protrusions and directional cell migration^25^. The hetero-oligomerization of Exo70 with the other exocyst subunits is also needed for the secretion of MMPs, including MMP-2 and MMP-9^18,24^. We thus analyzed whether Exo70 phosphorylation on Ser47, Ser59, and Ser89 influences its ability to form homopolymers or to bind other exocyst subunits. Co-IP assay showed that the self-interaction of Exo70 proteins was suppressed when they were co-expressed with ULK1 (Fig. 5a). The ability of Exo70 proteins to self-assemble increased when Ser47, Ser59, and Ser89 were mutated to alanine alone or in combination, and this effect was no longer able to be suppressed by the co-expression of ULK1 (Fig. 5a). Consistently, the single-point mutant S89D and the double mutants S47D/S89D and S59D/S89D partially inhibited the self-association of Exo70, whereas the triple mutant Exo70-3D was almost incapable of self-association (Fig. 5b, both Exo70-Flag and HA-Exo70 were mutated). The potential oligomerization of recombinant Exo70-WT and Exo70-3D was also examined by gel-filtration chromatography. Compared to Exo70-WT, the peak fraction of Exo70-3D shifted to the lower-molecular weight fractions (Fig. 5c, d). It was previously shown that oligomerization of Exo70 induces membrane protrusions on the cell surface^17,20,23^. We then investigated whether Exo70 phosphorylation by ULK1 affects membrane protrusion formation. Using fluorescence microscopy, we compared the formation of membrane protrusions after expressing GFP-tagged Exo70 variants in MDA-MB-231 cells. As shown in Fig. 5e, f, Exo70-WT and Exo70-3A induced much more protrusions than Exo70-3D.

Using IP assay, we also found that the interactions of Exo70-3D with several other exocyst subunits, including Sec6, Sec8, and Exo84, were markedly decreased (Fig. 5g). We also tested the effect of Exo70 or its mutants on MMPs secretion in MDA-MB-231 cells using zymography. Cells transfected with Exo70-WT showed a significant increase of MMP-9 secretion, whereas cells expressing Exo70-3D showed mildly reduced MMP-9 secretion when compared to the vector control (Fig. 5h).

### Exo70-Ser89 phosphorylation in human breast cancer tissues

ULK1 expression was negatively associated with breast cancer progression and is a prognostic marker for breast cancer^7^. Since our data showed that Exo70 phosphorylation by ULK1 inhibited breast cancer metastasis, we investigated the relationship between ULK1 expression and the phosphorylation levels of Exo70 in clinical breast cancer tissues. For this aim, we attempted to generate antibodies that bind specifically to one of these three phosphorylation sites. However, only the anti-phospho-Exo70 (Ser89) antibody was successfully obtained (Supplementary Fig. 4a). We also tested whether this antibody was specific at the cellular level, using overexpressed and endogenous Exo70 proteins. Plasmids expressing Exo70-WT, Exo70-Ser47A, or Exo70-Ser89A was transfected alone or together with ULK1 into 293T cells, and the whole-cell lysates were subjected to western blot analysis. The antibody recognized ULK1-induced phosphorylation of both endogenous Exo70 and overexpressed Exo70-WT and Exo70-Ser47A, but not Exo70-Ser89A (Supplementary Fig. 4b). Moreover, ULK1-induced phosphorylation level of endogenous Exo70, detected using this antibody, was greatly attenuated after knocking down endogenous Exo70 (Supplementary Fig. 4c).

The relationship between ULK1 expression and the phosphorylation level of Exo70 on Ser89 in clinical breast cancer tissues was then examined using a commercial ULK1 antibody and our anti-phospho-Exo70-Ser89 antibody. As shown in Fig. 5i, the phosphorylation level of Exo70 on Ser89 was significantly decreased in human invasive breast cancer tissues as compared to the adjacent normal tissues, and was positively correlated with the expression level of ULK1.

### Exo70 phosphorylation by ULK1 is suppressed by ERK1/2

**Fig. 6:**
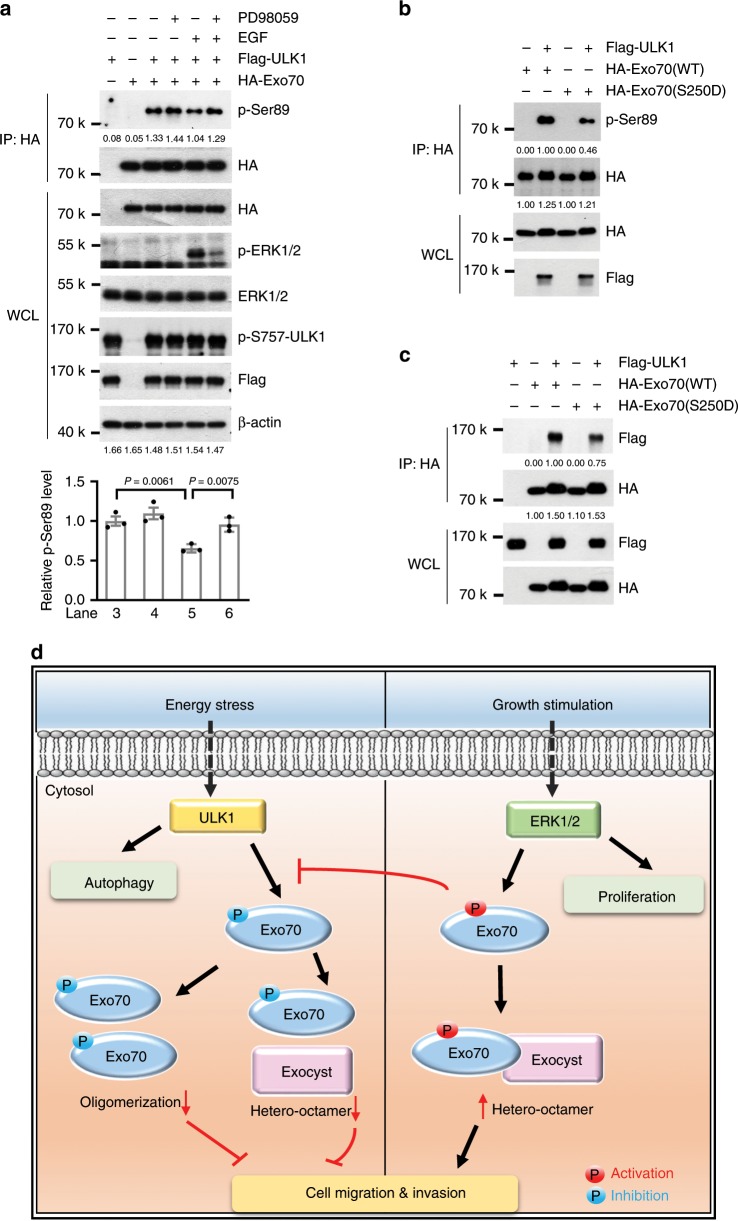
ULK1 phosphorylation of Exo70 was suppressed by ERK1/2 phosphorylation. **a** ULK1 phosphorylation of Exo70 on Ser89 was affected by EGF and PD98059 treatment. The 293T cells transfected with ULK1-Flag and/or HA-Exo70 as indicated were treated with EGF or PD98059. The cell lysates were then subjected to immunoprecipitation with anti-HA antibody. Phosphorylation of Exo70 at Ser89 was examined by the anti-pSer89 antibody and quantified by densitometry. Data represented the mean ± SEM (*n* = 3, three independent experiments), and the *P* values were analyzed by unpaired two-tailed Student’s *t* test. **b** ULK1 phosphorylation of Exo70 on Ser89 was lower than that of Exo70(S250D). The 293T cells were co-transfected with ULK1-Flag and HA-Exo70 or HA-Exo70(S250D), and then subjected to immunoprecipitation with anti-HA antibody. Phosphorylation of Exo70 at Ser89 was examined by the anti-pSer89 antibody. **c** The interaction of ULK1 with Exo70(S250D) was compared with that of ULK1 and wild-type Exo70 by co-immunoprecipitation assay. **d** Regulation of Exo70 by ULK1 and ERK1/2 under different growth condition. Upon energy stress, ULK1 was activated and phosphorylated Exo70 on Ser47, Ser59, and Ser89, subsequently inhibiting the self-oligomerization of Exo70 and its interaction with other exocyst subunits; thereafter, decreasing filopodial protrusions formation and MMP secretion, which led to the suppression of breast cancer cell migration and invasion. Under growth stimuli, activated ERK1/2 phosphorylated Exo70 on Ser250, which facilitates cell invasion via enhancing the interaction of Exo70 with other exocyst subunits and thus increasing invadopodia formation and MMP secretion (our published data^18^); additionally, Exo70 phosphorylation on Ser250 by ERK1/2 could also suppressed ULK1-induced phosphorylation of Exo70 to release metastasis inhibition.

We have previously shown that phosphorylation of Exo70 on Ser250 by ERK1/2 stimulates cell invasion by promoting the interaction of Exo70 with the other exocyst components, which in turn leads to enhanced invadopodia formation and MMP secretion^18^. We then asked whether ERK1/2 phosphorylation affects Exo70 phosphorylation by ULK1. As shown in Fig. 6a, phosphorylation of Exo70 on Ser89 by ULK1 was inhibited after epidermal growth factor (EGF) treatment; the inhibitory effect was attenuated by the addition of PD98059, an inhibitor of ERK1/2. Consistent with this observation, the level of ULK1-induced phosphorylation on Ser89 decreased in the Exo70(S250D) mutant, which was shown to mimic S250 phosphorylation by ERK^18^ (Fig. 6b). The S250D mutation also decreased the binding of Exo70 to ULK1 (Fig. 6c). These results suggest that ULK1 phosphorylation of Exo70 is inhibited by ERK1/2 phosphorylation of Exo70 on Ser250 in response to growth factor signaling.

## Discussion

ULK1 expression was recently linked to breast cancer metastasis^7^. In this study, we demonstrated that ULK1 suppressed the migration and invasion of human breast cancer cells. We have identified Exo70, a key component of the exocyst complex essential for cell motility and invasion^18,24^, as a direct ULK1 downstream phospho-substrate. ULK1 phosphorylation of Exo70 on Ser47, Ser59, and Ser89 led to the suppression of breast cancer cell migration and invasion. We have generated a specific anti-phospho-Exo70 (Ser89) antibody, and showed that Exo70 phosphorylation on Ser89 was significantly decreased in human breast cancer tissues compared to the paired adjacent normal tissues. The level of Exo70-Ser89 phosphorylation positively correlated with the expression level of ULK1. Together, these results demonstrated that ULK1 mediates the phosphorylation and inactivation of Exo70, resulting in the suppression of breast cancer cell motility and metastasis.

Mechanistically, we demonstrate that phosphorylation of Exo70 inhibits its homo-oligomerization and its interaction with other exocyst subunits, which are required for cell surface protrusion formation and MMP secretion during invasion^25^. The ULK1 phosphorylation sites, Ser47, Ser59, and Ser89, are located to the N terminus of Exo70, a region that was previously shown to be involved in its homo-oligomerization^25^. Our recent cryo-electron microscopy study in yeast indicates that the N-terminal CorEx motif of Exo70, which is conserved from yeast to human, is responsible for its interaction with Exo84, Sec10, Sec15, and other subunits to form the intact exocyst complex^29^. The prediction on the secondary structure showed an α-Helix exist in amino acids 5–102, which included the ULK1 phosphorylation sites^29^. The phosphorylation of Exo70 on these residues may bring in electrostatic charges or disrupt the proper conformation of α-helix that destabilize these interactions, thereby affecting the homo-oligomerization and the assembly of the exocyst complex.

Opposite to the inhibitory phosphorylation by ULK1, our previous work demonstrated that Exo70 can be phosphorylated by ERK1/2 in response to growth factors such as EGF, thereby promoting cell invasion^18^. Our present study demonstrates that Exo70 phosphorylation on Ser250 by ERK1/2 suppressed ULK1-mediated phosphorylation of Exo70. We propose a model that, under stress condition, ULK1 is activated and suppresses Exo70 to keep breast cancer cells dormant. However, under conditions such as growth factor stimulation, Exo70 is phosphorylated by ERK on S250. The phosphorylation of Exo70 on S250 not only promotes exocyst assembly but also blocks the inhibitory phosphorylation by ULK1, which may sustain cell invasion for metastasis (Fig. 6d).

Our finding provides a mechanism that regulates tumor cell invasion, and may potentially lead to the development of tools for prognosis and therapeutic treatment of breast cancer.

## Methods

### Cell culture

Human embryonic kidney cell line 293T (Cat. #GNHu17), human breast cancer cell lines MDA-MB-231 (Cat. #TCHu227), MCF-7 (Cat. #SCSP-531), and MDA-MB-453 (Cat. #TCHu233) were obtained from the Institute of Cell Biology, Shanghai, China. Human breast cancer cell lines Bcap-37 and BT-549 were kindly provided by Professor Bo-an Li (Xiamen University, Xiamen, Fujian, China). The 293T, MDA-MB-231, and MCF-7 cells were maintained in Dulbecco’s modified Eagle’s medium (DMEM, Gibco, Grand Island, NY, USA). MDA-MB-453 cells were maintained in Leibovitz’s L-15 medium (Gibco, Grand Island, NY, USA). Bcap-37 and BT-549 cells were maintained in RPMI-1640 medium (Gibco, Grand Island, NY, USA). All media were supplemented with 10% fetal bovine serum (FBS, HyClone, Logan, UT, USA), 100 U ml^−1^ penicillin and 100 μg ml^−1^ streptomycin (Life Technologies, Carlsbad, CA, USA). Cells were cultured at 37 °C in humidified incubator containing 5% CO_2_. All cell lines were identified by STR (Short Tandem Repeat) profiling by the source. Cells were expanded after being received and subsequently stored in liquid nitrogen. The storage vials were thawed for experiments and used in <2 months. All cell lines were confirmed negative for mycoplasma. For starvation condition, Earle’s balanced salt solution (EBSS), Hank’s balanced salt solution (HBSS), and DMEM medium sugarless were used.

### IP, western blot, and dot-blot analyses

Cells were lysed in ice-cold lysis buffer (50 mM Tris-HCl, pH 7.5, 150 mM NaCl, 100 mM NaF, 1% Triton X-100, 2 mM EDTA, 40 mM β-glycerolphosphate, 1 mM sodium orthovanadate, 1 μg ml^−1^ leupeptin, 1 μg ml^−1^ pepstin, 1 µg ml^−1^ aprotinin, and 1 mM phenylmethylsulfonyl fluoride), sonicated five times for 3 s each, and centrifuged at 16,000 × *g* for 15 min at 4 °C. The protein concentration of the supernatant was measured by BCA protein assay kit (Thermo Scientific, Rockford, IL, USA). For IP or co-IP, cell lysates were incubated with antibodies overnight at 4 °C. Protein A/G plus-agarose beads (Santa Cruz Biotechnology, Santa Cruz, CA, USA) were added into the lysates and incubated for 3 more hours. Beads were then washed three times in lysis buffer and then boiled. For western blotting, the boiled samples were run on sodium dodecyl sulfate-polyacrylamide gels (SDS-PAGE) and transferred onto Immobilon^TM^-P PVDF membranes (Merck Millipore, Billerica, MA, USA). After blocking with 5% skim milk in phosphate-buffered saline with 0.1% Tween-20 for 1 h, the membranes were incubated with antibodies. Immunoreactive bands were detected using Enhanced Chemiluminescence (ECL) system (Bio-Rad, Hercules, CA, USA). For dot blotting, 1–2 μl antigen was spotted onto a piece of membrane, left dry for 30 min or longer, and then blocked and incubated with antibody and the blots were detected as described above.

### Clinical tissue analysis

Clinical sample study was approved by the Medical Ethics Committee of Zhongshan Hospital Affiliated to Xiamen University in accordance with the Declaration of Helsinki and conducted with the informed consent of all patients. All breast tissue samples were obtained from the tissue bank of Zhongshan Hospital Affiliated to Xiamen University. All these five cases are invasive breast carcinomas that correspond to different subtypes. Case 2 and case 3 are triple-negative (ER-negative, PR-negative, and HER-2-negative); cases 1 and 5 are luminal A (ER- and PR-positive); case 4 is HER-2-enriched (ER- and PR-negative, HER-2-positive). Tissue samples were homogenized and lysed in ice-cold lysis buffer as described above and subjected to western blotting.

### GST pull-down assay

For GST pull-down assay, HA-ULK1 proteins were overexpressed in 293T cells and immunoprecipitated with anti-HA antibody and protein A/G plus-agarose beads as described in IP assay, and then eluted with HA peptide. Recombinant GST-Exo70 protein was expressed in *Escherichia coli*, and then purified and conjugated to glutathione sepharose, which was further incubated with the purified HA-ULK1 proteins in ice-cold lysis buffer at 4 °C for 3 h. Beads were then washed three times in lysis buffer and boiled, and the samples were then subjected to western blotting.

### In vitro phosphorylation assay

For in vitro phosphorylation assay, HA-ULK1, HA-ULK1(M92A), GST-Exo70, and its mutant variants were purified. The purified Exo70 proteins (without GST-tag) were released from the glutathionesSepharose beads by incubation with thrombin (Cat. #T4648, Sigma-Aldrich, St. Louis, MO, USA) overnight at 4 °C.

Prepared Exo70 or its mutants (without GST-tag) together with the HA-ULK1 or HA-ULK1(M92A) proteins, as mentioned above, were then added into the kinase buffer (25 mM HEPES, pH 7.4, 50 mM NaCl, 5 mM MgCl_2_, 1 mM dithiothreitol (DTT), 0.5 mg ml^−1^ bovine serum albumin, 200 μM ATP) and incubation at 30 °C for 1 h. Samples were then processed for western blotting.

### Transwell assay

Cells (5 × 10^4^) were plated on 8.0-μm pore size Boyden chambers (Millipore Corp., Billerica, MA, USA), either coated with bovine collagen I (Trevigen, Gaithersburg, MD, USA, for invasion assays) or uncoated (for migration assays), in serum-free medium containing 10 g l^−1^ bovine serum albumin. Medium containing 10% FBS was used as a chemoattractant in the lower chamber. After incubation at 37 °C for 12 h for migration assay and 18 h for invasion assay, the non-invaded or non-migrated cells were removed from the upper face of the transwell membrane using cotton swabs. The migrated or invaded cells were fixed with 4% paraformaldehyde and stained using 0.1% crystal violet in methanol, and then counted in randomly selected fields under a light microscope.

### Wound healing assay

Cells were grown to a confluent monolayer in a six-well tissue culture dish. Cell monolayers were wounded using a sterile p200 pipette tip, and then washed twice with phosphate-buffered saline (PBS) to remove cell debris and incubated in medium with 1% FBS. Cell migration was monitored under an inverted microscope equipped with a camera. The wound distance (width) at different time point was measured. The wound open (%) = wound distance/AWD_0_ × 100; AWD_0_: average wound distance at 0 h.

### In vivo tail vein metastasis assay

MDA-MB-231 cell sublines stably expressing luciferase (1 × 10^6^ cells per mouse) were injected into the tail vein of 5-week-old female nude mice. After 4 weeks, mice were imaged on the ventral side for luciferase expression via Caliper IVIS Lumina II. Five minutes before imaging, mice were injected intraperitoneally with 200 μl of 10 mg ml^−1^ d-luciferin (Promega, Cat. #E1605) in PBS and then imaged with a Caliper IVIS Lumina II Kinetic system. Total photon flux of the ventral thoracic area was measured and analyzed using the ROI tool in Living Image 4.4 software for lung metastasis. All animal experiments were performed in accordance with protocol approved by the Animal Care and Use Committee of Xiamen University.

### Extracellular matrix degradation assay

The coverslips (20 mm) were coated with 0.01% poly-l-lysine solution (Sigma-Aldrich, Cat. #P8920) and fixed with 0.5% glutaraldehyde for 15 min. The coverslips were inverted onto 80-μl droplets of gelatin matrix (0.2% gelatin and Alexa Fluor 488-labeled gelatin (Molecular Probes, Cat. #G-13186) at an 8:1 ratio) for 15 min and incubated in 5 mg ml^−1^ of NaBH_4_ (Sigma-Aldrich) for 15 min, followed by extensive washing with Dulbecco’s PBS (Gibco). Finally, the plate was incubated in DMEM for 1 h at 37 °C before adding the cells.

To examine the ability of cells to degrade matrix, 2 × 10^4^ MDA-MB-231 cells expressing HA-ULK1 or HA-ULK1(M92A) were plated onto the coverslips and incubated at 37 °C for 20 h, and processed for immunofluorescence. HA-ULK1 or HA-ULK1(M92A) were stained with mouse anti-HA (Cat. #H9658, 1:500), followed by Cy3-conjugated goat anti-mouse IgG secondary antibody (Cat. #115-165-003, 1:300), and F-actin was stained by anti-Phalloidin-iFuor 647 conjugate antibody (Cat. #ab176759, 1:300). The matrix degradation index was analyzed by Image J (National Institutes of Health, Bethesda, MD, USA). For quantification, the degradation level of each cell was measured as the area of the degraded zones normalized to the area of the entire field, and then the percentage of cells with different degradation levels was calculated.

### In-gel zymography

Cells were cultured in serum-free DMEM for 24 h, and the medium were collected and concentrated. Samples were then separated on an 8% SDS-PAGE gel containing 1 mg ml^−1^ gelatin. The gel was washed twice in washing buffer (2.5% Triton X-100 in H_2_O), incubated in developmental buffer (50 mM of Tris-HCl, pH 7.6, and 5 mM of CaCl_2_) at 37 °C for 48 h and stained with Coomassie Blue. The clear bands indicate where MMPs degraded gelatin. Gel loading was normalized to the total proteins.

### Gel filtration

Four hundred microliters (800 μg) of recombinant Exo70 or Exo70(3D) mutant was loaded onto a pre-equilibrated Superdex 200 10/300GL column and eluted with PBS containing 1 mM DTT at a flow rate of 0.5 ml min^−1^. Five hundred microliters of fractions were collected. Twenty microliters of each fraction was subjected to 10% SDS-PAGE and stained with Coomassie Blue.

### MS analysis of Exo70 phosphorylation

Exogenous Exo70-Flag, co-overexpressed with HA-ULK1 or the empty vector in 293T cells (cultured in full medium), was immunoprecipitated with anti-Flag antibody and protein A/G-agarose as mentioned in IP assay. Immunoprecipitated samples were run on SDS-PAGE and stained with Coomassie Blue. For liquid chromatography-mass spectrometer (LC-MS)/MS analysis, the Exo70 samples in the SDS-PAGE were excised and digested with trypsin overnight and the peptides were separated by a 120 min gradient elution at a flow rate of 0.30 µl min^−1^ with a Dionex Ultimate 3000 HPLC system (Thermo Fisher Scientific, Waltham, MA, USA), which is directly interfaced with a Q Exactive mass spectrometer (Thermo Fisher Scientific, Waltham, MA, USA). The analytical column was a home-made fused silica capillary column (75 µm ID, 150 mm length; Upchurch, Oak Harbor, WA, USA) packed with C-18 resin (300 Å, 5 µm, Varian, Lexington, MA, USA). Mobile phase consisted of 0.1% formic acid, and mobile phase B consisted of 80% acetonitrile and 0.1% formic acid. The Q Exactive mass spectrometer was operated in the data-dependent acquisition mode using the Xcalibur 2.1.2 software (Thermo Fisher Scientific, Waltham, MA, USA) and there was a single full-scan mass spectrum in the orbitrap (300–1800 *m*/*z*, 70,000 resolution), followed by 20 data-dependent MS/MS scans at 27% normalized collision energy (HCD). The MS/MS spectra from each LC-MS/MS run were searched against the Rat.fasta from Uniprot using an in-house Proteome Discoverer (Version PD1.4, Thermo Fisher Scientific, Waltham, MA, USA). The search criteria were as follows: two missed cleavage was allowed; phosphorylation was set as the variable modification. The PhosphoRS algorithm was used to calculate the probability of the phosphorylation sites. When the PhosphoRS probability was above 75%, the phosphorylation site was considered to be authentic.

### Statistics and reproducibility

All results were presented as means ± SEM. The statistical details including the definitions and value of *n* (e.g., number of experimental replicates, micrographs, cells, animals, etc.) were provided in the figures and corresponding figure legends. All gels and blots were representative of three independent experiments. Statistical significance was determined by two-tailed unpaired Student’s *t* test or one-way analysis of variance using GraphPad Prism 6 (GraphPad Software), or Kruskal–Wallis test using the SPSS 19 software.

### Other methods

Detailed descriptions of the other methods used in this study, including antibodies and reagents, plasmids, transient transfection, lentivirus-mediated stable overexpression or knockdown, real-time PCR, immunofluorescent staining, MTT assay, and tumor xenograft assay are provided in the Supplementary Materials and Methods.

### Reporting summary

Further information on research design is available in the Nature Research Reporting Summary linked to this article.

## Supplementary information

Supplementary Information

Reporting Summary

## Data Availability

The source data underlying Figs. 1–6 and Supplementary Figs. 1–4 are provided as a Source Data file. All the other data supporting the findings of this study are available within the article and its Supplementary information files and from the corresponding author upon reasonable request.
