# Supplementary material for: ULK1 phosphorylates Exo70 to suppress breast cancer metastasis
Source: Nat Commun. 2020 Jan 8;11:117. doi: 10.1038/s41467-019-13923-7 (PMC6949295; doi:10.1038/s41467-019-13923-7)
Supplement: Supplementary file 1 — Supplementary Information [file 41467_2019_13923_MOESM1_ESM.pdf]

Supplementary Information for

ULK1 Phosphorylates Exo70 to Suppress Breast Cancer Metastasis

Liyuan Mao, Yan-yan Zhan, Bin Wu, Qiang Yu, Liang Xu, Xiaoting Hong, Linhai Zhong, Panying Mi, Li Xiao, Xinquan Wang, Hanwei Cao, Wenqing Zhang, Binbin Chen, Jingzhou Xiang, Kunrong Mei, Ravi Radhakrishnan, Wei Guo, and Tianhui Hu

This file includes:

Supplementary Figures 1–4

Supplementary Materials and Methods

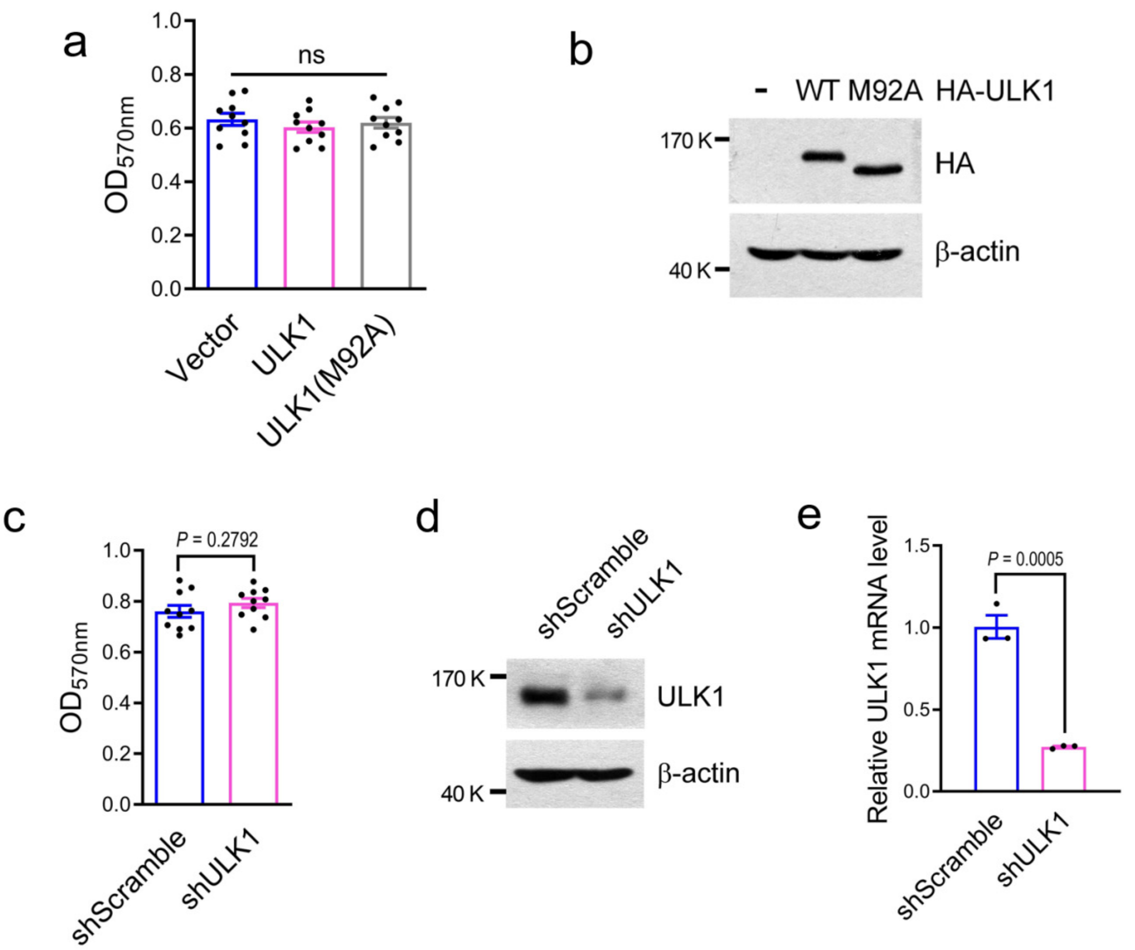

Supplementary Figure 1 | ULK1 inhibits the migration and invasion of breast cancer cells. (**a**) Overexpression of ULK1 or ULK1(M92A) did not affect the growth or survival of MDA-MB-231 cells during the observed time period in Fig. 1b-c as determined by MTT assay (n = 10 biologically independent samples). (**b**) The expression levels of transfected HA-ULK1 and HA-ULK1(M92A) plasmids were examined by western blotting. (**c**) Knocking down ULK1 did not affect the growth or survival of MCF-7 cells within 48 hrs as determined by MTT assay (n = 10 biologically independent samples). (**d-e**) ULK1 knockdown efficiency in MCF-7 cells was examined by western blotting (**d**) and real-time PCR (**e**, n = 3 biologically independent samples). Data represented the mean ± SEM. *P* values were analysed by unpaired two-tailed Student's *t*-test, ns nonsignificant *vs* control.

	S47 S59 S89
Human (isoform 1,2,4,5,6,7) 35-102	SDQLTKNMVSILSSFESRLMKLENSIIPVHKQTENLQRLQENVEKTLSCLDHVISYYHVASDTEKIIR
Human (isoform 3) 1-61	MVSILSSFESRLMKLENSIIPVHKQTENLQRLQENVEKTLSCLDHVISYYHVASDTEKIIR
Rat 35-102	SDQLTKNMVSILSSFESRLMKLENSIIPVHKQTENLQRLQENVEKTLSCLDHVISYYHVASDTEKIIR
Mouse 35-102	SDQLTRNMVSILSSFESRLMKLENSIIPVHKQTENLQRLQENVEKTLSCLDHVISYYHVASDTEKIIR
Zebrafish 35-102	SDQLTKGMVSILSSFESRLMQLENSIIPVHKQTENLQRLQENVDKTLSNMDHVISYYHVAKDTDKIIR
Sumatran Orangutan 35-102	SDQLTKNMVSILSSFESRLMKLENSIIPVHKQTENLQRLQENVEKTLSCLDHVISYYHVASDTEKIIR
Cricetulus griseus 50-117	SDQLTKNMVSILSSFESRLMKLENSIIPVHKQTENLQRLQENVEKTLSCLDHVISYYHVASDTEKIIR
Cattle 35-102	SDQLTKNMVSILSSFESRLMKLENSIIPVHKQTENLQRLQENVEKTLSCLDHVISYYHVASDTEKIIR
Xenopus (Silurana) tropicalis 35-102	SDQLTNNMVSILSSFESRLMKLENSIIPVHKQTETLQRLQENVDRTLSCLDHVISYYHVATETERIIK
Chimpanzee 35-102	SDQLTKNMVSILSSFESRLMKLENSIIPVHKQTENLQRLQENVEKTLSCLDHVISYYHVASDTEKIIR

Supplementary Figure 2 | The motif surrounding Ser47, Ser59 and Ser89 of human Exo70 (isoform 1, 2, 3, 4, 5, 6, 7) is evolutionarily highly conserved in eukaryotic cells.

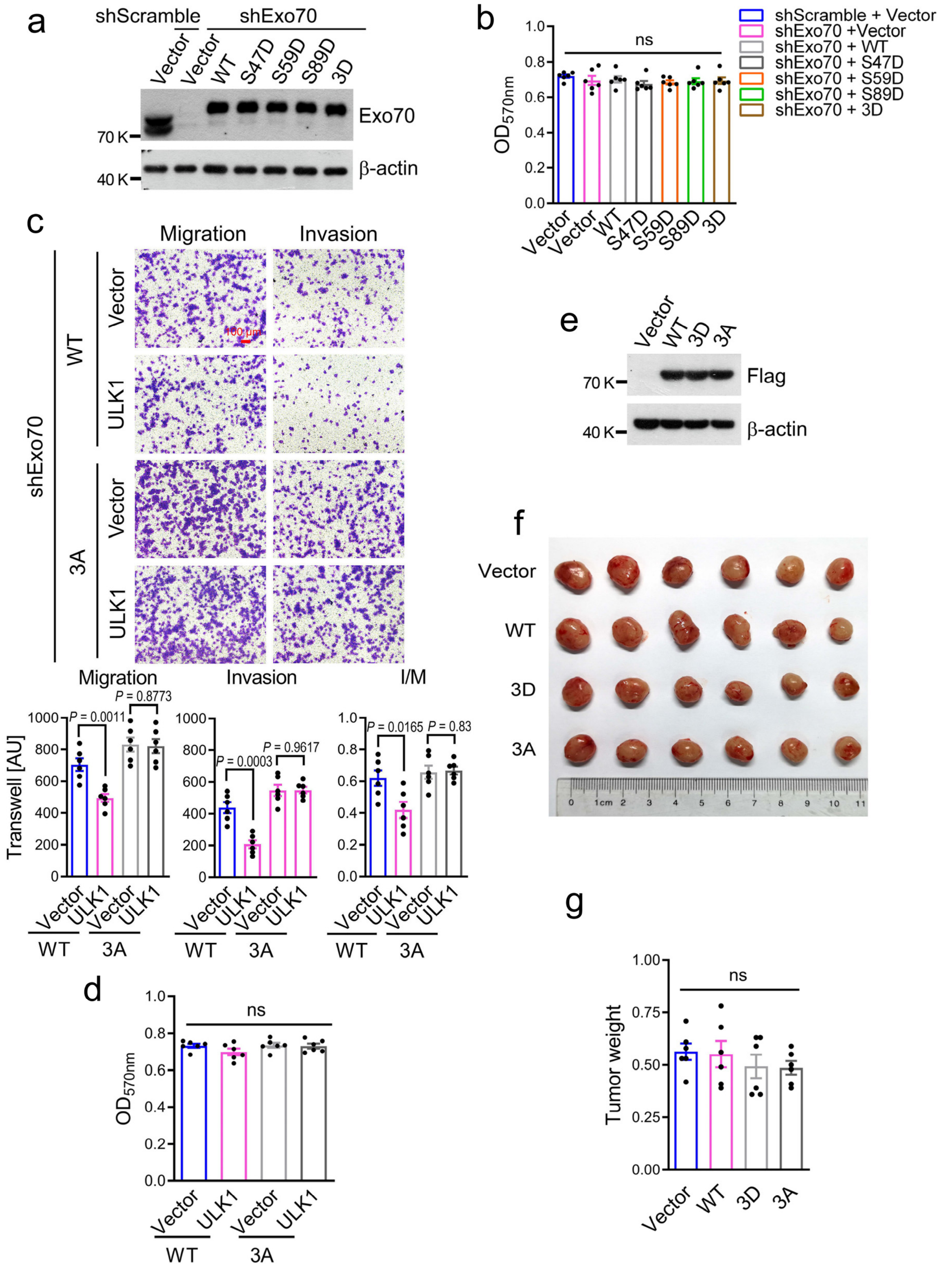

Supplementary Figure 3 | ULK1 inhibits migration and invasion of breast cancer cells. (**a**) The expression levels of transfected Exo70-Flag or Exo70 mutant variants in Fig. 4a-d were examined by western blotting. (**b**) Effects of Exo70 and its mutant variants on the proliferation

or survival of MDA-MB-231 cells within 40 hrs was detected by MTT assay (n = 6 biologically independent samples). (**c**) Phospho-deficient Exo70 mutants rescued ULK1-mediated inhibition of cell migration and invasion as indicated by transwell assays. Representative images (Scale bar: 100 μm) (**upper**) and cell counts (**lower**) were shown (n = 6 biologically independent samples). AU: arbitrary unit. I/M: ratio of invasion (I) to migration (M). (**d**) Proliferation and survival of MDA-MB-231 cells in different groups during the period of assay in supplementary Fig. 3c was detected by MTT assay (n = 6 biologically independent samples). (**e**) The expression levels of transfected Exo70-Flag or its mutant variants in Fig. 4e-f were examined by western blotting. (**f-g**) Effects of Exo70 and its mutants on the proliferation of breast cancer cells *in vivo* indicated by tumor xenograft assay. The diameter (**f**) and weight (**g**) of tumors in these groups were detected and compared (n = 6 per group). Data represented the mean ± SEM, ns nonsignificant *vs* vector control by unpaired two-tailed Student's *t*-test.

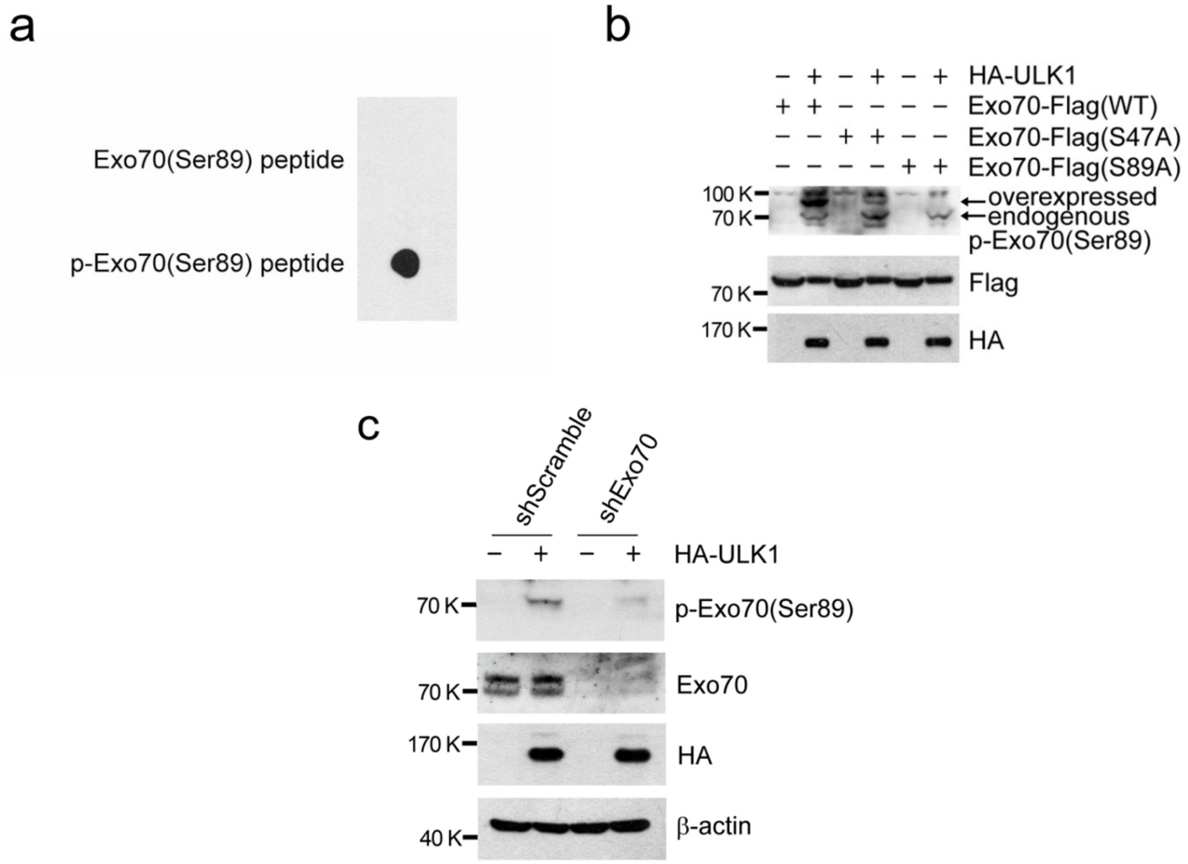

Supplementary Figure 4 | Verification of the anti-phospho-Exo70 (Ser89) antibody. (**a**) The specificity of the anti-phospho-Exo70 (Ser89) antibody was examined by dot blotting. (**b-c**) The specificity of the anti-phospho-Exo70 (Ser89) antibody was tested at the cellular level with western blot analysis, by overexpressing Exo70 or its mutants (**b**) or knocking down endogenous Exo70 (**c**) in 293T cells.

Supplementary Materials and Methods

Antibodies and reagents

Rabbit anti-Exo70 (Cat. #ab118792, 1:1000 for western blot (WB), 1:100 for Immunoprecipitation (IP)), rabbit anti-pSer/Thr (Cat. #ab17464, 1:1000 for WB), anti-Phalloidin-iFluor 647 conjugate (Cat. #ab176759), rabbit anti-ULK1 (Cat. #ab240916) and mouse anti-β-actin antibodies (Cat. #ab3280, 1:10000 for WB) were purchased from Abcam, Cambridge, MA, USA. Mouse anti-Exo70 (Cat. #sc-365825), anti-Cortactin-Alexa Fluor® 647 conjugate (Cat. #sc-55579AF647), and mouse anti-Myc (Cat. #sc-40, 1:400 for WB) antibodies were purchased from Santa Cruz Biotechnology, Santa Cruz, CA, USA. Rabbit anti-phospho-ULK1(Ser757) (Cat. #6888, 1:1000 for WB), rabbit anti-ULK1 (Cat. #8408, 1:1000 for WB, 1:100 for IP), anti-phospho-ERK1/2 (Cat. #4376, 1:1000 for WB) and anti-ERK1/2 (Cat. #4695, 1:1000 for WB) antibodies were purchased from Cell Signaling Technology, Beverly, MA, USA. Mouse anti-Flag (Cat. #F3165, 1:5000 for WB, 1:500 for IP) and anti-HA (Cat. #H9658, 1:20000 for WB, 1:1000 for IP) antibodies were purchased from Sigma-Aldrich, St. Louis, MO, USA. FITC-conjugated goat anti-mouse IgG secondary antibody (Cat. #115-095-003), FITC-conjugated goat anti-rabbit IgG secondary antibody (Cat. #111-095-003), Cy3-conjugated goat anti-rabbit IgG secondary antibody (Cat. #111-165-003), Cy3-conjugated goat anti-mouse IgG secondary antibody (Cat. #115-165-003) were purchased from Jackson ImmunoResearch Laboratories, Inc., West Grove, PA, USA. Recombinant human epidermal growth factor (EGF) (Cat. #PHG0311) was purchased from Thermo Fisher Scientific, Waltham, MA, USA. Rapamycin (Cat. #R0395) and PD98059 (Cat. #P215) was purchased from Sigma-Aldrich, St. Louis, MO, USA.

Plasmids

The pCDNA3.3-3×HA-ULK1(mus) (kindly provided by Sheng-cai Lin, Xiamen University, Xiamen, Fujian, China) and pCMV14-Exo70(rattus)-3×Flag were subcloned into vectors as needed, such as pCMV10-3×Flag, pCDNA3.3-3×HA, pCDNA3.3-Myc, pGEX-4T-1, pLV-puro and pCW57.1-puro. The point mutations in Exo70 and ULK1 were constructed using the QuickChange Mutagenesis Kit (Stratagene, La Jolla, CA, USA) according to the manufacturer's instructions and verified by sequencing.

Transient transfection, stable overexpression and knockdown

For transient transfection, polyethylenimine (PEI, Cat. #23966, Polysciences, Warrington, PA) was used to transfect 293T cells at a final concentration of 10 μM. Transfected cells were harvested after 24 hrs for further analysis.

For lentivirus-mediated stable overexpression or knockdown, lentiviruses were generated by transfecting 293T cells with the lentiviral vector (pLL3.7 for knockdown, pLV-puro for overexpression, pCW57.1-puro for Doxycycline inducible HA-ULK1 or HA-ULK1(M92A) expression in MDA-MB-231 cells) and packaging plasmids (pHR and pCMV-VSV-G) using Lipofectamine 2000 transfection reagent (Invitrogen, Carlsbad, CA, USA). Viral supernatants were collected 48 hrs after transfection, centrifuged at 3000 g for 15 min, and filtered through 0.45-μm filters (Millipore, Billerica, MA, USA). Freshly plated MDA-MB-231 and MCF-7 cells were infected with the packaged lentivirus and selected by puromycin (2 $\mu g \cdot ml^{-1}$). The expression of HA-ULK1 or HA-ULK1(M92A) in MDA-MB-231 cells were induced by

Doxycycline (1 μg·ml^{-1}) before transwell assay and extracellular matrix degradation assay.

The shRNA sequences targeting human ULK1 was 5′-GCACAGAGACCGTGGGCAA-3′. The shRNA sequences targeting human Exo70 was 5′-TGCAGGAGAATGTTGAGAA-3′. The shRNA control (scramble) sequence was 5′-GGCTACGTCCAGGAGCGCACC-3′. Oligonucleotides (Invitrogen, Guangzhou, China) were annealed and inserted into the pLL3.7 vector.

Real-time PCR

Total RNA was extracted with Trizol reagent (TaKaRa, Dalian, China) and reverse-transcribed to cDNA using Primescript™ RT reagent kit (TaKaRa). The analyses were performed using the SYBR Green I fluorescent dye (SYBR® Premix Ex Taq™ II, TaKaRa, Dalian, China) and the StepOnePlus™ real- time PCR system (Applied Biosystems, Australia). The thermal cycling consisted of an initial pre-degeneration at 95 °C for 30 sec, followed by 40 cycles of de-naturation at 95 °C for 5 sec and annealing/extension at 60 °C for 30 sec. Primers used were listed as follows:

ULK1: 5′-CCCAGAGGAGACCCTCATG-3′ (forward)

5′-CAATGGCAGTCTGTAGGCC-3′ (reverse)

GAPDH: 5′-TGCACCACCAACTGCTTAGC-3′ (forward)

5′-GGCATGGACTGTGGTCATGAG-3′ (reverse)

Immunofluorescence staining

After culture medium was discarded, cells were fixed immediately in 4%

paraformaldehyde in PBS for 10 min at room temperature and then permeabilized with 0.2% Triton X-100 in PBS for 10 min on ice. Coverslips were blocked in 5% BSA and 0.02% Triton X-100 in PBS for 30-60 min. Mouse anti-Exo70 (Cat. #sc-365825) and rabbit anti-ULK1 (Cat. #ab240916) antibodies were diluted at 1:200 dilution in PBS containing 5% bovine serum albumin and incubated overnight at 4 ºC. Following incubation, coverslips were washed five times for 5 min in PBS containing 1.5% bovine serum albumin and then incubated with a 1:200 dilution of FITC-conjugated goat anti-mouse IgG secondary antibody (Cat. #115-095-003), and a 1:300 dilution of Cy3-conjugated goat anti-rabbit IgG secondary antibody (Cat. #111-165-003) and anti-Cortactin-Alexa Fluor® 647 conjugate antibody (Cat. #sc-55579AF647) for 1 hr at room temperature. The nuclei were stained with 1 mmol·L^{-1} DAPI (Sigma-Aldrich, St. Louis, MO, USA) for 5 min. Images of cells were obtained using Zeiss LSM 880 Laser Scanning Confocal Microscope.

MTT Assay

Cells were seeded at a density of 6,000 per well into 96-well plates and cultured for 24 or 48 hrs as needed in the medium containing 10% FBS. After that, MTT (Sigma-Aldrich, St. Louis, MO, USA) was added at a concentration of 50 μg per well, and the cells were cultured for another 3 hrs. The medium was then replaced with 150 μl DMSO to dissolve the formazan crystals. The number of viable cells was evaluated by measuring the absorbance at an OD of 570 nm (Abs) using a spectrophotometer.

Tumor xenograft assay

MDA-MB-231 cell lines stably expressing Exo70-WT, Exo70-3D, Exo70-3A or a control vector (2×10^6 cells per mouse) were subcutaneously injected into 5-week-old female nude mice. Four weeks later, mice were sacrificed, and the diameter and weight of tumors in these groups were measured. All animal experiments were performed in accordance with protocol approved by the Animal Care and Use Committee of Xiamen University.
