# Supplementary material for: ULK1 phosphorylates Exo70 to suppress breast cancer metastasis
Source: Nat Commun. 2020 Jan 8;11:117. doi: 10.1038/s41467-019-13923-7 (PMC6949295; doi:10.1038/s41467-019-13923-7)
Supplement: Supplementary file 2 — Reporting Summary [file 41467_2019_13923_MOESM2_ESM.pdf]

nature research

Corresponding author(s): Wei Guo, Yanyan Zhan, Tianhui Hu

Last updated by author(s): Jul 28, 2019

Reporting Summary

Nature Research wishes to improve the reproducibility of the work that we publish. This form provides structure for consistency and transparency in reporting. For further information on Nature Research policies, see Authors & Referees and the Editorial Policy Checklist .

Statistics

For all statistical analyses, confirm that the following items are present in the figure legend, table legend, main text, or Methods section.

n/a	Confirmed	
[ ]	[x]	The exact sample size (*n*) for each experimental group/condition, given as a discrete number and unit of measurement
[ ]	[x]	A statement on whether measurements were taken from distinct samples or whether the same sample was measured repeatedly
[ ]	[x]	The statistical test(s) used AND whether they are one- or two-sided<br>*Only common tests should be described solely by name; describe more complex techniques in the Methods section.*
[x]	[ ]	A description of all covariates tested
[x]	[ ]	A description of any assumptions or corrections, such as tests of normality and adjustment for multiple comparisons
[ ]	[x]	A full description of the statistical parameters including central tendency (e.g. means) or other basic estimates (e.g. regression coefficient) AND variation (e.g. standard deviation) or associated estimates of uncertainty (e.g. confidence intervals)
[ ]	[x]	For null hypothesis testing, the test statistic (e.g. *F*, *t*, *r*) with confidence intervals, effect sizes, degrees of freedom and *P* value noted<br>*Give P values as exact values whenever suitable.*
[x]	[ ]	For Bayesian analysis, information on the choice of priors and Markov chain Monte Carlo settings
[x]	[ ]	For hierarchical and complex designs, identification of the appropriate level for tests and full reporting of outcomes
[x]	[ ]	Estimates of effect sizes (e.g. Cohen's *d*, Pearson's *r*), indicating how they were calculated

Our web collection on statistics for biologists contains articles on many of the points above.

Software and code

Policy information about availability of computer code

Data collection	Zeiss Zen 2.3.0, Thermo Fisher Xcalibur 2.1.2, Caliper Living Image 4.3.1, Unicorn 5.20
Data analysis	Statistical analyses were performed with GraphPad Prism and SPSS 19 software. Gray values for western blotting were analyzed using Photoshop software. Immunofluorescence analyses were performed using Zeiss Zen 2.3.0 software. Transwell assays were performed with Image-Pro Plus software. The matrix degradation index was analyzed by Image J software.

For manuscripts utilizing custom algorithms or software that are central to the research but not yet described in published literature, software must be made available to editors/reviewers. We strongly encourage code deposition in a community repository (e.g. GitHub). See the Nature Research guidelines for submitting code & software for further information.

Data

Policy information about availability of data

All manuscripts must include a data availability statement. This statement should provide the following information, where applicable:

- Accession codes, unique identifiers, or web links for publicly available datasets
- A list of figures that have associated raw data
- A description of any restrictions on data availability

The data that support the findings of this study are available from the corresponding authors upon reasonable request.

Field-specific reporting

Please select the one below that is the best fit for your research. If you are not sure, read the appropriate sections before making your selection.

Life sciences study design

All studies must disclose on these points even when the disclosure is negative.

Sample size	Samples size for each experiment is indicated in the figures or corresponding figure legends. For animal experiments, in each group at least 6 mice were used in order to obtain statistical significance.
Data exclusions	One mouse with premature death during tail vein metastasis assay was excluded. The remaining animals were all included in the studies.
Replication	Experiments were repeated at least 3 times to ensure reproducibility except otherwise indicated in figure legends.
Randomization	The allocation of cells/mice to different treatments was completely random.
Blinding	For cellular and mouse studies, the experiments were performed in a blinded fashion whenever possible. For example, analyses of mouse models (measuring tumor weight and luminescence intensity) were performed in a blinded fashion; individuals performing the assays were not aware of the treatment groups until the data analyses were completed.

Reporting for specific materials, systems and methods

We require information from authors about some types of materials, experimental systems and methods used in many studies. Here, indicate whether each material, system or method listed is relevant to your study. If you are not sure if a list item applies to your research, read the appropriate section before selecting a response.

Materials & experimental systems

n/a	Involved in the study	
☐	☒	Antibodies
☐	☒	Eukaryotic cell lines
☒	☐	Palaeontology
☐	☒	Animals and other organisms
☒	☐	Human research participants
☒	☐	Clinical data

Methods

n/a	Involved in the study	
☒	☐	ChIP-seq
☒	☐	Flow cytometry
☒	☐	MRI-based neuroimaging

Antibodies

Antibodies used	See supplementary materials and methods, chapter "Antibodies and reagents". All the antibody used are commercial and validated excepted for the p-Ser89(Exo70) antibody, which has been purified and validated (Supplementary Figure 4).
Validation	Validation statement for antibody is provided on the manufacture's website. Antibodies were further validated by using positive and negative controls in our studies.

Eukaryotic cell lines

Policy information about cell lines

Cell line source(s)	HEK293T, MDA-MB-231, MCF-7 and MDA-MB-453 were obtained from the Institute of Cell Biology and ATCC. Bcap-37 and BT-549 were kindly provided by Professor Bo-an Li (Xiamen University, Xiamen, Fujian, China).
Authentication	All of the cell lines used were authenticated by STR profiling.
Mycoplasma contamination	Mycoplasma testing was regularly conducted to assure that all cells used were mycoplasma free.
Commonly misidentified lines (See ICLAC register)	No commonly misidentified cell lines were used for this study.

Animals and other organisms

Policy information about studies involving animals; ARRIVE guidelines recommended for reporting animal research

Laboratory animals	5-week-old female nude mice were housed under standard specific-pathogen-free (SPF) conditions.
Wild animals	Study did not involve wild animals.
Field-collected samples	No samples were collected in Field.

Ethics oversight	All animal experiments were performed in accordance with protocols approved by the Institution Animal Care and Ethics Committee of Xiamen University.

Note that full information on the approval of the study protocol must also be provided in the manuscript.
